# Thermomechanical Properties of Virgin and Recycled Polypropylene—High-Density Polyethylene Blends

**DOI:** 10.3390/polym15214200

**Published:** 2023-10-24

**Authors:** Hannah Jones, Jake McClements, Dipa Ray, Colin S. Hindle, Michail Kalloudis, Vasileios Koutsos

**Affiliations:** 1 School of Engineering, Institute for Materials and Processes, The University of Edinburgh, Sanderson Building, King’s Buildings, Edinburgh EH9 3FB, UK; 2 School of Engineering, Newcastle University, Merz Court, Claremont Road, Newcastle upon Tyne NE1 7RU, UK; 3 School of Engineering and The Built Environment, Edinburgh Napier University, Merchiston Campus, 10 Colinton Road, Edinburgh EH10 5DT, UK; 4 Impact Laboratories Ltd. (Impact Solutions), Impact Technology Centre, Fraser Road, Kirkton Campus, Livingston EH54 7BU, UK

**Keywords:** polypropylene, polyethylene, blends, recycled, thermomechanical properties

## Abstract

This paper provides evidence and discusses the variability in the thermomechanical behaviour of virgin and recycled polypropylene/high-density polyethylene blends without the addition of other components, which is sparse in the literature. Understanding the performance variability in recycled polymer blends is of critical importance in order to facilitate the re-entering of recycled materials to the consumer market and, thus, contribute towards a circular economy. This is an area that requires further research due to the inhomogeneity of recycled materials. Therefore, the thermal and mechanical properties of virgin and recycled polypropylene/high-density polyethylene blends were investigated systematically. Differential scanning calorimetry concludes that both the recycled and virgin blends are immiscible. Generally, recycled blends have lower overall crystallinity and melting temperatures compared with virgin blends while, remarkably, their crystallisation temperatures are compared favourably. Dynamical mechanical analysis showed little variation in the storage modulus of recycled and virgin blends. However, the alpha and beta relaxation temperatures are lower in recycled blends due to structural deterioration. Deterioration in the thermal and mechanical properties of recycled blends is thought to be caused by the presence of contaminants and structural degradation during reprocessing, resulting in shorter polymeric chains and the formation of imperfect crystallites. The tensile properties of recycled blends are also affected by the recycling process. The Young’s modulus and yield strength of the recycled blends are inferior to those of virgin blends due to the deterioration during the recycling process. However, the elongation at break of the recycled blends is higher compared with the virgin blends, possibly due to the plasticity effect of the low-molecular-weight chain fragments.

## 1. Introduction

Plastic waste is a major environmental issue, with only 9% of the world’s plastics being recycled [1]. Polyolefins, encompassing polypropylene (PP) and polyethylene (PE), are the main components in municipal waste due to their abundant use in commodity applications as they possess good mechanical properties and processability, in addition to having high availability and low manufacturing costs [2,3,4,5]. The complete separation of PP and PE during mechanical waste recovery is uneconomical due to their close densities and structural similarity; therefore, PP and PE usually remain mixed [6,7,8]. During the mechanical recycling process, PP and PE undergo irreversible thermomechanical degradation processes such as chain scission, which lowers the mechanical properties of the recycled PP (rPP) and recycled PE (rPE) compared with the virgin polymers [9]. 

Blends of PP and PE are of great commercial interest as they have the potential to reduce the deficient characteristics of PP and PE, such as low impact resistance at low temperatures and poor environmental stress cracking resistance, respectively [6,10,11]. However, blends of PP: High-Density PE (HDPE) and PP: Low-Density PE (LDPE) are thermodynamically immiscible, resulting in poor material performance due to their phase-separated morphology and low interfacial adhesion between the phases [12,13]. The mechanical performance of immiscible blends are dependent upon the blend components’ crystallisation behaviour and final blend morphology [14,15,16]. Several factors are important for morphology development during polymer processing such as composition, viscosity ratio of the components, interfacial properties, crystallinity and processing conditions [6,14,17,18,19,20,21,22]. Several studies have reported the mechanical properties, crystallisation behaviour and morphology of PP:PE blends [6,7,11,22,23,24,25,26,27,28].

Although it is important to determine and understand the mechanical and thermal behaviour of the virgin PP (vPP): virgin polyethylene (vPE) blends in order to optimise the properties of recycled blends, comparative studies are also important. A comparison of the thermomechanical properties of vPP:vHDPE and rPP:rHDPE blends over a wide range of compositions is lacking as the literature mainly focuses on ternary systems (e.g., PP:HDPE and compatibilisers/co-polymers/other polymers/fillers [5,29,30,31,32]). It is important to understand the variability in performance of rPP:rHDPE blends before the addition of further components. 

Studies into the crystallinities of rPP, rHDPE and rPP:rHDPE blends have found that crystallinity is affected by degradation mechanisms during the recycling process [33,34,35]. Interestingly, rPP crystallinity has been found to be higher than that of vPP by several authors [33,35,36]. da Costa et al. [35] suggested that the higher value of crystallinity of rPP compared with vPP was caused by a decrease in the molecular weight, which resulted in an increase in chain mobility. Increased chain mobility improved the ability of chains to fold into thicker lamella and, hence, an increased crystallisation rate and crystallinity. Therefore, it is important to understand the variation in crystallinity in rPP:rHDPE blends in order to optimise the mechanical performance of the recyclates.

Research has been undertaken in the literature to understand the effect of recycling cycles on the mechanical properties of PP, PE and their blends. For example, Aurrekoetxea et al. [33] subjected PP to 10 successive injection moulding cycles at 200 °C and found that the degree of crystallinity increased with each cycle. This caused an increase in Young’s modulus and yield stress. On the other hand, Oliveira et al. [37], who subjected PP to seven successive cycles at 175–190 °C, observed a decrease in Young’s modulus and yield stress after the third cycle that was attributed to a reduction in tie molecules between the crystalline and amorphous phases. Conflicting observations by Aurrekoetxea et al. [33] and Oliveira et al. [37] for the Young’s modulus and yield stress of rPP could be due to differences in the processing methodology. Aurrekoetxea et al. [33] used injection moulding, whereas Oliveira et al. [37] opted for a single screw extruder followed by compression moulding. This highlights the importance of the reprocessing methodology but also demonstrates the difficulty of comparing the performance of recycled materials in the literature. Furthermore, chemical analysis studies of recycled PE and PP have revealed their variability, degradation and the presence of impurities and contaminants [38,39,40].

PE can be subjected to a higher number of extrusion cycles before any deterioration in the mechanical properties is observed. Jin et al. [41] found no significant change in crystallinity and, hence, in the mechanical properties of LDPE up to the 40th extrusion cycle. However, a decrease in crystallinity was observed between the 40 and 50th cycles, either caused by short side branches in the backbone chain or side groups, or by crosslinking. Oblak et al. [42] subjected HDPE to 100 consecutive extrusion cycles at 220–270 °C. They found that chain branching and chain scission, which occurred up to the 60th cycle, resulted in a decrease in crystallinity and Young’s modulus. However, crystallinity and Young’s modulus remained stable after the 60th extrusion cycle due to crosslinking. After the 100th cycle, the Young’s modulus of the rHDPE had only reduced by 20% compared with that of the vHDPE. 

Studies have been carried out to understand the mechanical properties of PP:PE blends subjected to recycling cycles [9,26,43,44,45,46,47]. Saikrishnan et al. [43] reported that recycling affected the melt flow behaviour of PP:LDPE blends but found that the tensile properties were not substantially affected (subjected to up to five recycling cycles). Interestingly, PP underwent chain scission on each recycling cycle but the overall properties of the blend were maintained. However, they only investigated the PP:LDPE up to 10 wt% of LDPE. The literature is typically limited in the blend composition range investigated. However, due to the variability in the waste streams, it is important to understand the mechanical properties for all blend compositions without the addition of a third component initially. This would enable the recycling industry to be reactive to changes in waste stream composition in different locations and batches and enable more recyclate to re-enter the market. Therefore, this paper aims to understand the variability in thermomechanical properties for virgin and recycled PP:HDPE blends.

This study reports the thermal and mechanical properties of vPP:vHDPE and rPP:rHDPE blends through differential scanning calorimetry (DSC), dynamical mechanical analysis (DMA) and tensile testing. This comparative study enables the mapping of not only the challenges but also the potentially unique opportunities of the recycled systems. The recycling industry is looking to improve the plastic circular economy by obtaining recycled commingled waste blends with desirable end-use properties acceptable for commercial applications but at low cost.

## 2. Experimental Section

### 2.1. Materials

PP (Moplen EP440G), supplied by LyondellBasell (London, UK), had a melt flow index (MFI) of 1.3 g 10 min^−1^ and density of 900 kg m^−3^. HDPE (HDPE, K46-06-185), supplied by Ineos (Grangemouth, UK), had an MFI of 4.2 g 10 min^−1^ and a density of 946 kg m^−3^. Post-consumer rPP and rPE were supplied by Impact Solutions Recycled (Livingston, UK). The rPE was mainly composed of HDPE, but small quantities of LDPE were present. rPP and rHDPE had MFIs of 15 and 1.5 g 10 min^−1^, respectively. As shown by the MFI values, the grades of virgin and recycled PP and HDPE used are quite different; therefore, the properties are not directly comparable. The comparisons made through the study are more general between virgin and recycled grades. 

Virgin and recycled blends of different compositions of PP and HDPE (P10, P20, P25, P40, P50, P60, P75, P80 and P90) were prepared, where P denotes PP and the number corresponds to the percentage composition by weight of PP in the blend PP:HDPE. The pure 100 weight percentage (wt%) PP and HDPE will be denoted as PP and HDPE, respectively. To denote virgin or recycled, the symbols of v and r will be used, respectively, before the blend composition, e.g., virgin P10 would be represented as vP10. 

### 2.2. Preparation

#### Extrusion and Injection Moulding

vPP and vHDPE were in the form of pellets, whereas rPP and rPE were in the form of flakes. Blends were prepared using a lab scale Haake MiniCTW twin screw extruder (Karlsruhe, Germany) for 5 min with feeder and mixing speeds of 50 rpm and 100 rpm, respectively. The conical screws were 4–15 mm in diameter, 109.4 mm in length and co-rotate. The barrel temperature was 180–185 °C. Molten blends were transferred to the Haake MiniJet injection moulder (Karlsruhe, Germany), where the cylinder temperature was 210 °C, mould temperature was 35 °C, injection pressure was 50 MPa and hold-on pressure time was 10 s. The ISO 527-2-1BA [48] and 557–2296 moulds were used for the dog-bone-shaped and DMA rectangular samples, respectively. 

### 2.3. Characterisation

#### 2.3.1. DSC

The melting and crystallisation behaviour of the vPP:vHDPE and rPP:rHDPE blends were evaluated using a Perkin Elmer DSC 8000 (Waltham, MA, USA). The instrument was calibrated using an indium sample. Approximately 5–6 mg of the sample was scanned under a nitrogen atmosphere. Samples were exposed to the following thermal cycle: heated from 25 to 200 °C at 10 °C min^−1^, isothermal at 200 °C for 5 min, cooled from 200 to 25 °C at 10 °C min^−1^, isothermal at 25 °C for 2 min and heated from 25 to 200 °C at 10 °C min^−1^. The melting temperature *T*_m_ and enthalpy of fusion $∆H_{f}$ were obtained from the first heating ramp. The crystallisation temperature *T*_c_ was taken from the cooling ramp.

The degree of crystallinity was calculated by Equation (1),

(1) $$\%\;\mathrm{Crystallinity}\;=\frac{{∆H}_{f}^{\mathrm{obs}}}{{∆H}_{f}^{0}}\times 100$$

where ${∆H}_{f}^{\mathrm{obs}}$ is the observed enthalpy of fusions for the individual PP and HDPE peaks, and ${∆H}_{f}^{0}$ is the 100% crystalline HDPE or PP, which are 287 and 207 J g^−1^, respectively [6]. ${∆H}_{f}^{\;PP}$ and ${∆H}_{f}^{\;HDPE}$ were taken from the first heating ramp to calculate the crystallinity. The thermal history of the sample was erased after the first heating ramp [49]. However, very little difference was found when comparing the crystallinity obtained from the first and second heating ramps for the virgin and recycled blends (Tables S1 and S2). 

#### 2.3.2. DMA 

DMA was used to determine the viscoelastic properties of the virgin and recycled blends. A Triton DMA (Leicestershire, UK) in dual cantilever mode at a frequency of 1 Hz was used. A temperature sweep from −50 to 150 °C at a heating rate of 5 °C min^−1^ was implemented. Sample dimensions were approximately 45 mm (l) × 10 mm (w) × 2.7 mm (d). A minimum of three samples were tested, and the average and standard deviation were calculated for each blend ratio. 

The dynamic response was given as the elastic (storage modulus, *E*′), viscous (loss modulus, *E*″) and damping (tan delta, tanδ) components. The glass transition (*T*_g_) and transition relaxation processes can be seen as changes in the *E*″ or tanδ traces [50]. The tanδ trace was used to quote the *T*_g_ and other relaxation peaks present [51].

#### 2.3.3. Tensile Testing 

Tensile properties were determined using an Instron Tensile Machine (Buckinghamshire, UK) with a crosshead speed of 5 mm min^−1^ and a 10 kN load cell. Tensile properties were carried out at ambient temperature in accordance with the ISO 527-2 standard. Young’s modulus was determined using a Zwick Roell Tensile Machine with a video-extensometer. A crosshead speed of 1 mm min^−1^, gauge length of 25 mm and a 10 kN load cell were used. A minimum of five samples were tested, and the average and standard deviation were calculated. 

The “rule of mixtures” was used to predict the Young’s modulus of the virgin and recycled PP:HDPE blend samples compared to the experimental data. The rule of mixtures was calculated by Equation (2),

(2) $$E_{\mathrm{Blend}}=W_{\mathrm{PP}}E_{\mathrm{PP}}+W_{\mathrm{HDPE}}E_{\mathrm{HDPE}}$$

where *E*_Blend_ is the Young’s modulus of the polymer blend; *W*_PP_ and *E*_PP_ are the weight fraction and Young’s modulus of PP, respectively; and *W*_HDPE_ and *E*_HDPE_ are the weight fraction and Young’s modulus of component HDPE, respectively [52]. The experimental Young’s modulus of the virgin and recycled homogenous PP and HDPE systems was used for the virgin and recycled *E*_PP_ and *E*_HDPE_, respectively. 

## 3. Results and Discussion

### 3.1. Thermal Properties of Virgin and Recycled PP:HDPE Blends

**Figure 1:**
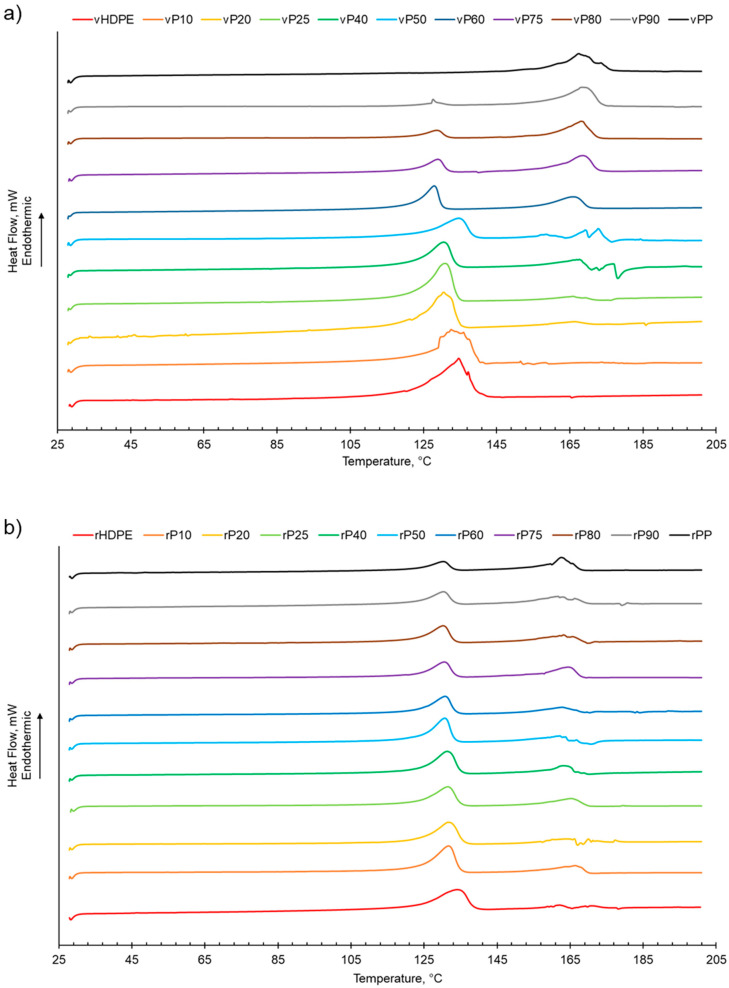
The melting behaviour of PP:HDPE blends obtained from DSC: (**a**) vPP:vHDPE blends and (**b**) rPP:rHDPE blends.

The melting behaviour of both rPP:rHDPE and vPP:vHDPE blends presented two separate peaks assigned to PP and HDPE, suggesting that the blends were immiscible (Table 1 and Figure 1). The two melting peaks present in the rPP and rPE indicated that contaminants were present due to the challenge of complete separation of PP and PE during the recycling process [26,29,53]. Little variation in the *T*_m_ of virgin and recycled PP, HDPE and their blends suggested that the blending of PP and HDPE did not significantly alter the *T*_m_ of PP and HDPE (Table 1, Figure S1) [6,29]. In some cases, the rPP:rHDPE blends had lower *T*_m_ than the respective vPP:vHDPE blends, indicating structural deteriorations of the polymeric components during the mechanical recycling process [54], which could imply that less-perfect crystallites formed [55].

**Figure 2:**
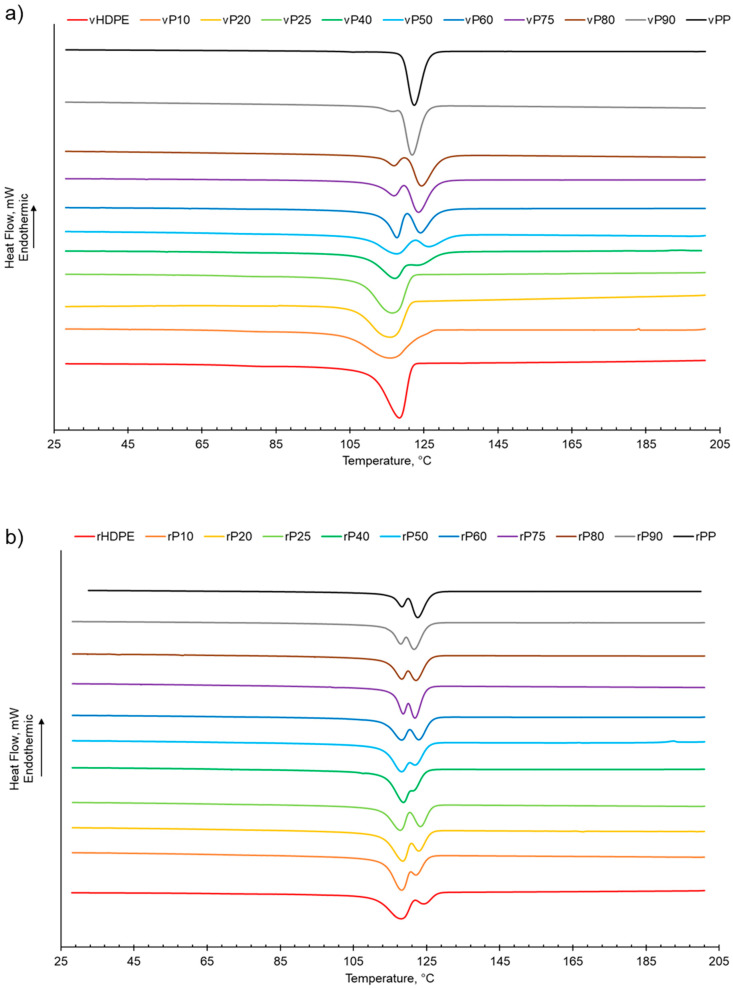
The crystallisation behaviour of PP:HDPE blends obtained from DSC: (**a**) vPP:vHDPE blends and (**b**) rPP:rHDPE blends.

As shown in Table 1 and Figure S2, increasing the PP wt% in the virgin or recycled PP:HDPE blends results a decrease in the enthalpy of crystallisation of HDPE while PP generally presents an increasing trend. This is in agreement with Jose et al. [6], who suggested that the decrease in enthalpy of crystallisation could be attributed to the differing rates of crystallisation for PP and HDPE and the resulting size of crystallites. PP crystallises at a slower rate compared with HDPE, which enables the formation of large spherulites. The large spherulites in PP liberate less energy during crystallisation compared with the smaller crystallites in HDPE [6]. The crystallisation behaviour of semi-crystalline polymers is more complex compared with their melting behaviour due to the numerous factors that can affect the phase structure, such as polymer composition and distribution, intra- and inter-molecular interactions, and processing conditions [56]. The presence of a second semi-crystalline material also complicates the crystallisation behaviour [8,57]. Typically, PP and HDPE crystallise separately and at different rates. HDPE has a quicker nucleation and growth rate compared with PP due to the HDPE’s flexible chain and limited intermolecular interactions [6,26,56]. In PP, crystallisation is hindered by the bulky methyl groups on the polymer chain backbone [56]. There was little variation observed in the crystallisation temperature (*T*_c_*)* for the recycled and virgin blends (Figure 2, Table 1 and Figure S3). Two crystallisation peaks were observed for rPP:rHDPE blends at the approximate individual PP and HDPE crystallisation temperatures. Phase separation is caused by the PP and HDPE crystals growing at different rates. Crystallisation peaks were observed in the rPP and rPE due to the presence of PE and PP contaminants, respectively. One crystallisation peak was observed for vPP:vHDPE blends up to vP25, suggesting co-crystallisation and/or partial miscibility. However, upon increasing the PP wt% further, two peaks were observed at the approximate individual PP and HDPE crystallisation temperatures, suggesting an onset of independent crystallisation and incompatibility. There is literature reporting a single crystallisation peak for vPP:vHDPE blends over a wide composition range. Lin et al. [27] and Sutar et al. [30] suggested that the addition of HDPE affected the PP crystallisation rate, resulting in one crystallisation peak. Jose et al. [6], who studied a range of PP:HDPE blends, reported only one crystallisation temperature, which possessed an intermediary *T*_c_ value between the *T*_c_ values of PP and HDPE. Aumunate et al. [26] found a single crystallisation peak for vPP:vHDPE blends caused by the merging of the vPP and vHDPE peaks due to their close *T*_c_. However, they suggested that bimodal behaviour was present at higher vHDPE contents due to the presence of a slight shoulder peak. 

**Figure 3:**
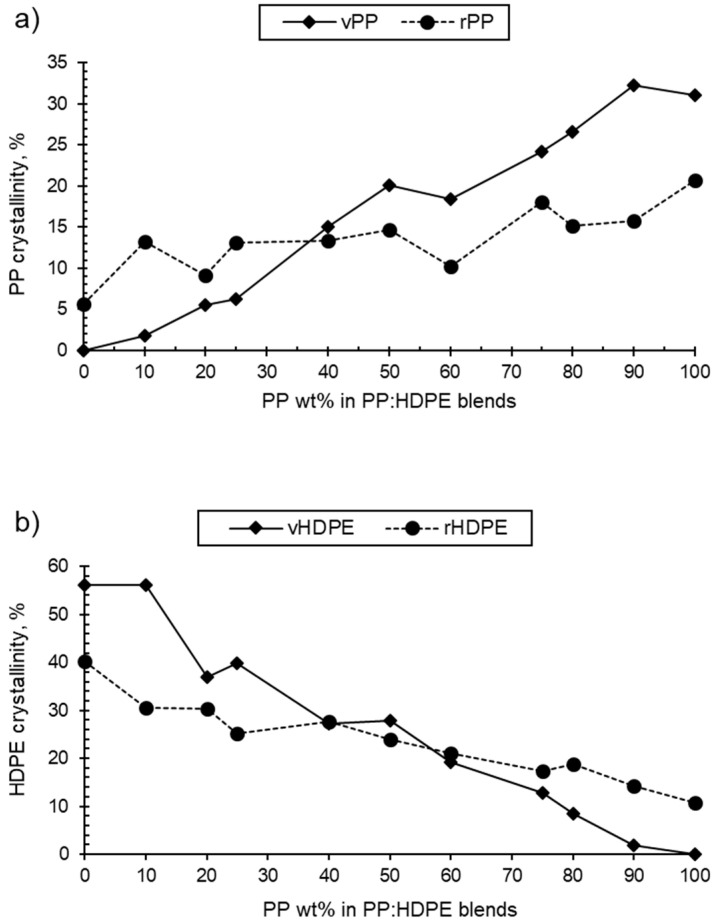
Variation of (**a**) virgin and recycled PP crystallinity in PP:HDPE blends and (**b**) virgin and recycled HDPE crystallinity in PP:HDPE blends.

As the PP wt% increased for both the rPP:rHDPE and vPP:vHDPE blends, the PP crystallinity in the blend increased and the HDPE crystallinity decreased (Table 1 and Figure 3). rPP crystallinity was higher than vPP crystallinity in HDPE up to the P25 blend. rHDPE crystallinity was higher than vHDPE crystallinity from P60 to PP. The crystallinity is affected by the presence of PP and HDPE contaminants in the recycled materials. The quantity of other plastic contaminants will be dependent on the waste stream composition and the quality of sorting at the material recycling facility [58,59]. Thermomechanical degradation, which occurs during recycling, results in an increase in polydispersity caused by the presence of shorter polymeric chains [60]. Shorter polymeric chains form crystals more easily compared with long chains due to their low degree of entanglement, which may lead to an increase in crystallinity [61]. On the other hand, the crystallinities of rPP and rHDPE were found to be lower than the vPP and vHDPE crystallinities for blends between P40 and PP, and HDPE and P50, respectively. The presence of other plastics, varying chains lengths and branching, and impurities such as oxidative moieties and additives can lead to the formation of imperfect crystallites and a heterogeneous crystalline morphology, hence reducing crystallinity [58,62]. Therefore, determining the exact cause of (enhanced or reduced) crystallinity in recycled blends is a challenge. It has to be noted that the *T*_g_ values of PP and HDPE were not observed in the thermograms as they are located below the starting temperature of the DSC thermograms.

### 3.2. Mechanical Properties of Virgin and Recycled PP:HDPE Blends

#### 3.2.1. DMA Measurements of Virgin and Recycled PP:HDPE Blends

**Figure 4:**
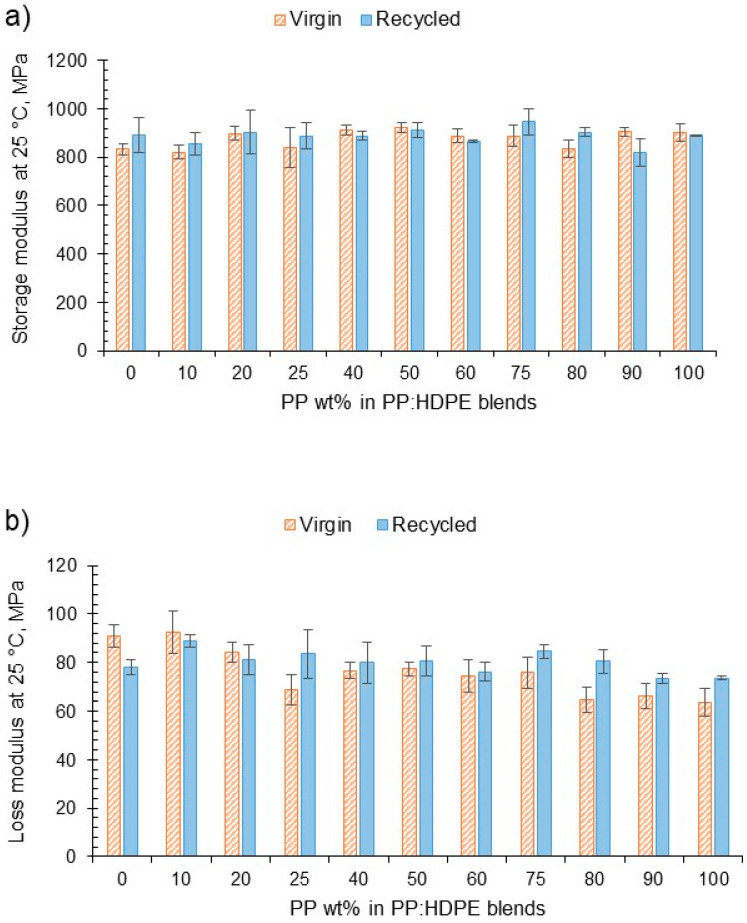
(**a**) The variation in storage modulus at 25 °C for vPP:vHDPE and rPP:rHDPE blends and (**b**) the variation in loss modulus at 25 °C for vPP:vHDPE and rPP:rHDPE blends.

DMA was used to determine the viscoelastic response of the blends as a function of temperature. *E*′ indicates the relative dynamic stiffness of the material and *E*″ indicates the ability to dissipate energy (Figure 4). No large variation was observed in *E*′ at different blend compositions for both the virgin and recycled blends. However, there was a decreasing trend for the loss modulus for the virgin blends. As the PP wt% increased, the vPP:vHDPE blend’s *E*″ decreased, whereas the rPP:rHDPE blends did not show such an obvious decrease in *E*″ with variation in composition. Interestingly, Fang et al. [47], who investigated the storage and loss moduli of rPP:rPE blends without the addition of a compatibiliser or filler, found an increase in moduli with rPP content. For example, the rP60 blend presented an *E*′ at 40 °C, which was twice that of the rP45 blend. They concluded that an increase in stiffness occurs with an increase in PP wt%. The difference in the temperature at which the moduli were taken, different manufacturing processes and MFI of the rPE and rPP could account for the differences observed. Structural deteriorations caused by the recycling process can introduce flexibility and mobility due to the shorter chains. However, impurities can act as fillers in the recycled materials, imposing a mechanical restraint that increases the stiffness [63]. 

**Figure 5:**
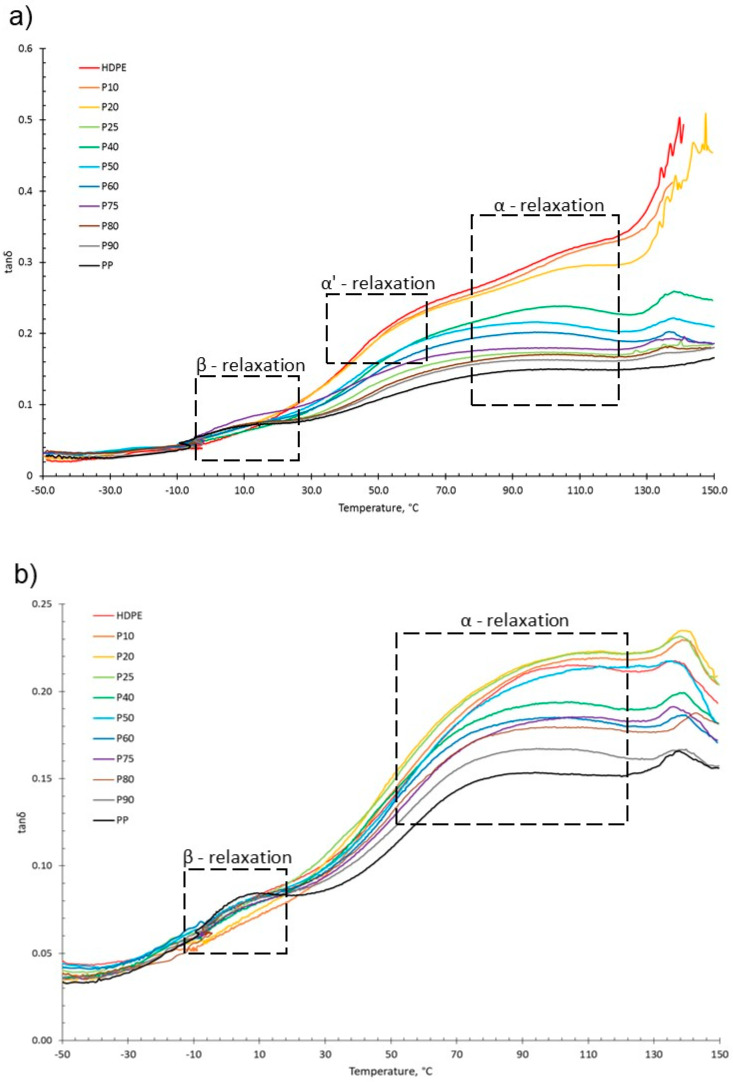
tanδ graphs as a function of temperature obtained from DMA of (**a**) vPP:vHDPE blends and (**b**) rPP:rHDPE blends with the relaxation regions highlighted.

**Figure 6:**
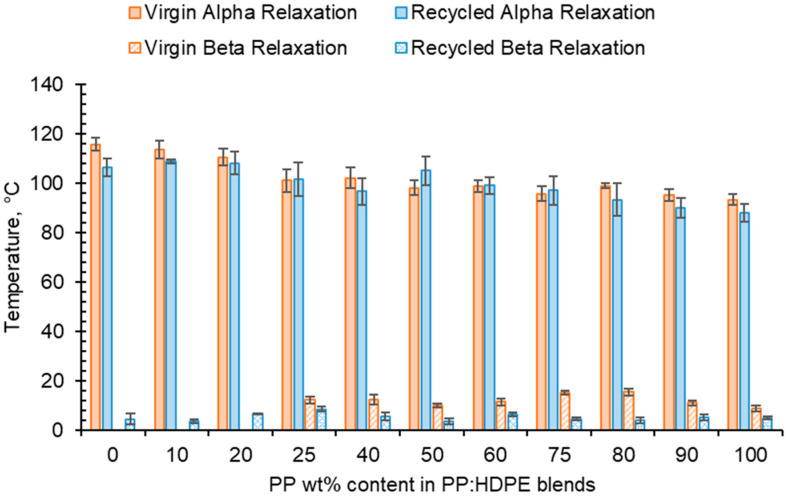
Comparison of *T*_α_ and *T*_β_ taken from tanδ traces for virgin and recycled PP:HDPE blends.

For both virgin and recycled PP, HDPE and their blends, as temperature increased, *E*′ decreased and *E*″ increased, shown in the tanδ vs. temperature graphs presented in Figure 5. This is due to material softening and the beginning of relaxation processes with increasing temperature [64]. HDPE and PP exhibit three relaxation processes: alpha (α), beta (β) and gamma (γ) [51,65]. Both the virgin and recycled PP:HDPE blends exhibited α and β relaxation processes in the tanδ vs. *T* graphs, as shown in Figure 5. γ relaxation was not observed as it typically occurs below −100 °C, which is outside the experimental temperature range. γ relaxation is associated with the motions of the side chain groups attached to the main chain in the amorphous region [65]. α relaxation is associated with the crystalline region, where the CH_2_ groups within the crystallites have vibrational and re-orientation motion. The chains are flexible and freely rotating [65,66,67]. The rHDPE and rPP alpha relaxation temperatures (*T*_α_) were lower compared with the vHDPE and vPP, respectively, possibly caused by imperfect crystallite formation due to recycling (Figure 5 and Figure 6, see also Table S1) [55]. The higher *T*_α_ of HDPE compared with PP could be due to HDPE’s higher crystallinity and amount of crystalline domains compared with PP [68]. The *T*_α_ of the virgin blends decrease as the PP wt% increases, with a similar trend observed in the recycled blends. The *T*_α_ are intermediary between the *T*_α_ of PP and HDPE. As suggested by Karaagac et al. [32], the observed relaxation temperatures of the blends are likely following the rule mixtures, and caution must be taken before suggesting partial miscibility at the interface due to the observation of a single peak. As the *T*_α_ of PP and HDPE are close in value, it is possible that there is an overlap in the peaks causing the blend to have a broad *T*_α_ peak.

HDPE exhibits an additional relaxation process, α′, which is associated with the crystalline region and partially overlaps into the α region [51]. α′ is observed in vHDPE at approximately 40–50 °C (Figure 5a). As the vHDPE content in the virgin blends decreases, α′ decreases in prominence. α′ is not observed in the rHDPE, possibly due to the recycling process, which causes the formation of imperfect crystallites and the presence of contaminants, thus decreasing the peak prominence.

β relaxation is associated with the motion of the branches in the amorphous region and is connected to the *T*_g_ [63,66,69]. The PP β relaxation temperature (*T*_β_) is the *T*_g_. There are many opposing viewpoints surrounding where the *T*_g_ of PE is: (a) in the β region just below 0 °C, (b) in the region of −81 °C and (c) in the γ region below −100 °C [65,70]. The magnitude of the *T*_β_ is dependent on the amount of amorphous domains, as the relaxation occurs in the amorphous domain. The *T*_β_ in HDPE may not always be observed due to the low proportion of amorphous domains compared with crystalline domains. Additionally, tie molecules between the crystalline and amorphous domains restrict the complete relaxation of amorphous chains [63,67]. The *T*_β_ observed will be that of vPP as the vHDPE *T*_β_ is not seen (Figure 5). The vPP *T*_β_ is not visible in the vP10 and vP20 blends due to the small magnitude of the relaxation. The *T*_β_ of PP becomes visible at 12.4 °C for vP25. The *T*_β_ is present in the rHDPE due to the presence of PP impurities, which cannot be completely removed in the recycling process [53]. The *T*_β_ of the recycled blends were lower than the virgin blends and had little variation. The recycling process results in a decrease in molecular weight. The presence of the low-molecular-weight chains causes an increase in free volume and reduced chain packing [71]. An increase in free volume lowers the *T*_β_ as less thermal energy is required for chain mobility. 

#### 3.2.2. Tensile Measurements of Virgin and Recycled PP:HDPE Blends

**Figure 7:**
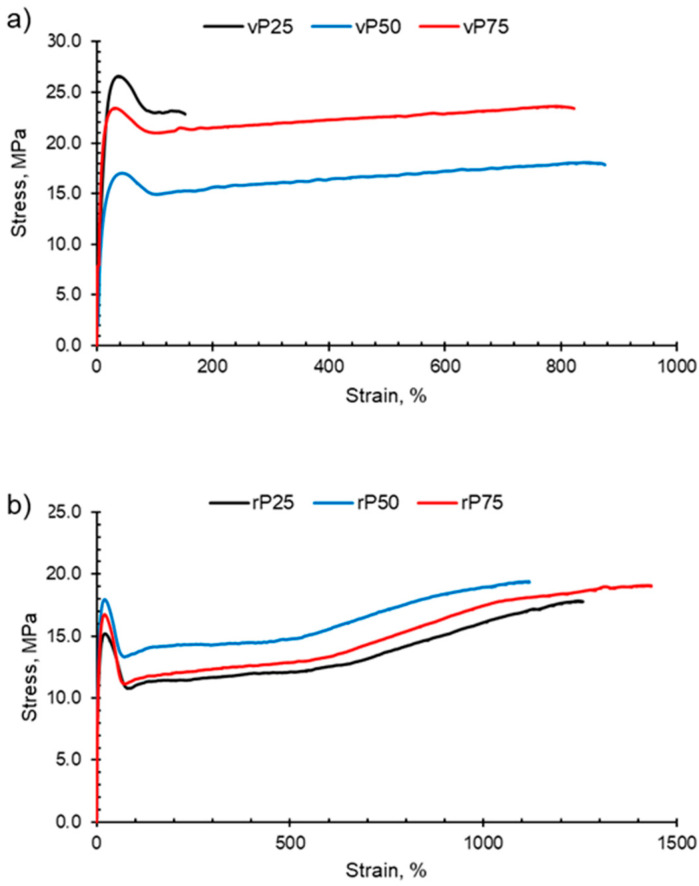
Examples of typical stress–strain curves obtained for PP:HDPE blends. The blends P25, P50 and P75 have been shown for (**a**) vPP:vHDPE blends and (**b**) rPP:rHDPE blends.

PP, HDPE and their blends undergo macroscopic deformation during a tensile test and typically exhibit strain hardening and a ductile fracture, as shown in Figure 7. Initially, the polymers undergo elastic deformation; however, as the force applied continues to increase, the polymer sample reaches the yield point and enters the region of plastic deformation. At the yield point, a small neck forms within the gauge section and the polymer chains align in the direction of elongation. Continuing beyond the yield point, the virgin and recycled PP, HDPE and their blends exhibit the strain-hardening phenomenon. Strain hardening occurs when there is resistance to deformation and the neck region propagates and extends, which is termed necking. The polymer chains continue to orientate and align in the direction of elongation, which results in an increase in the strength of the plastic. Necking continues until fracture. 

**Figure 8:**
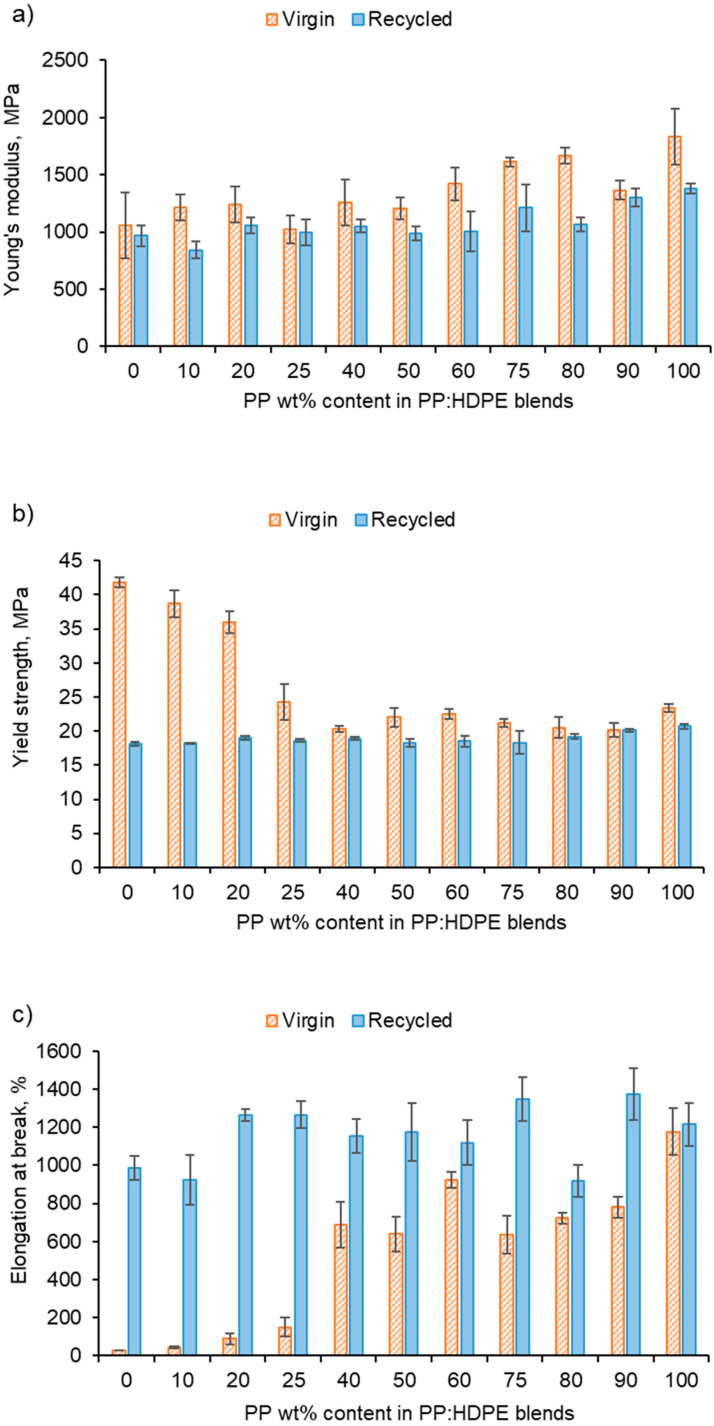
Tensile properties of vPP:vHDPE and rPP:rHDPE blends: (**a**) Young’s modulus, (**b**) yield strength and (**c**) elongation at break.

The recycled blends exhibited deteriorated tensile properties compared with the virgin blends in terms of Young’s modulus and yield strength (Figure 8). The Young’s modulus values of rPP and rHDPE were lower than those of the virgin polymers due to structural deterioration caused by the recycling process [60,72]. However, above rP75, the rPP:rHDPE blend’s yield strength values approach the virgin blend’s values. Studies have found that yield strength increases with crystallinity and lamellar thickness, with little or no effect of molecular weight [73]. The crystallinity of the recycled blends is lower compared with the virgin blends, and the recycling process results in the formation of imperfect crystals (which has been discussed in the thermal properties section), thus causing a reduction in the yield strength. Generally, there was little variation in the yield strength for the rPP:rHDPE blends.

The vHDPE demonstrated unexpected behaviour in yield strength (42 MPa) and elongation at break (29%) (Figure 8). vHDPE did not show typical necking behaviour and a brittle fracture was observed. No change in crystallinity was found by DSC when comparing the crystallinity of the HDPE before and after extrusion and injection moulding. During the injection moulding process, the polymer melt is exposed to a strong shear and elongational flow in which the chains are stretched and become highly orientated [74]. Flow-induced crystallisation increases HDPE’s crystallisation rate and forms a highly orientated shish-kebab structure, which improves the strength of HDPE [75]. Lei et al. [76] found no necking behaviour when vHDPE was blended with 4% ultra-high molecular weight PE prepared by twin screw extruder and dynamic injection moulding. An increase in the tensile strength in the flow direction was observed from 23 to 76 MPa, which was caused by the formation of a web-like shish-kebab morphology and chain orientation. Therefore, the high chain orientation of the vHDPE could result in an interlocking of the shish-kebab to form a rigid structure, which affected the yield strength and elongation at break up to the vP25 blend (Figure 8) [76,77,78]. The rHDPE did not exhibit the same unexpected behaviour as vHDPE in its yield strength and elongation at break. This is most likely due to the presence of lower-molecular-weight chains caused by the recycling processing, which have a reduced packing ability and degree of orientation. Additionally, the presence of micro-voids can result in a decrease in compatibility between polymeric components [79]. 

The comparison between the elongation at break for the virgin and recycled blends presents interesting results (Figure 8c). It was expected that recycled blends would have a lower elongation at break compared with the virgin blends due to the structural deterioration during recycling causing a reduction in molecular weight [80]. For example, Fang et al. [47] found that with the addition of rPP up to 15 wt% in a PP:PE blend, the elongation at break decreased, and with over 30 wt%, the elongation at break reached a minimum. However, the longer elongation at break observed for the recycled blend could be due to the presence of lower-molecular-weight polymer chains caused by the recycling process [81]. It is possible that the low-molecular-weight polymer chains locate at the interface between PP and HDPE phases and lower the interfacial tension [81]. Additionally, the lower-molecular-weight chains increase the capability of molecules sliding over each other, resulting in an increase in deformability [80]. The vHDPE up to vP25 presented extremely low elongation at break and samples exhibited brittle fractures. The data sheet provided by Ineos suggests an elongation at break value of 800% for vHDPE at 2 in min^−1^. As discussed, this behaviour could be due to the formation of a very rigid crystalline structure for vHDPE (and up to vP25), which would explain the brittle fracture observed. Due to this behaviour, a comparison between the virgin and recycled blends is more complex.

**Figure 9:**
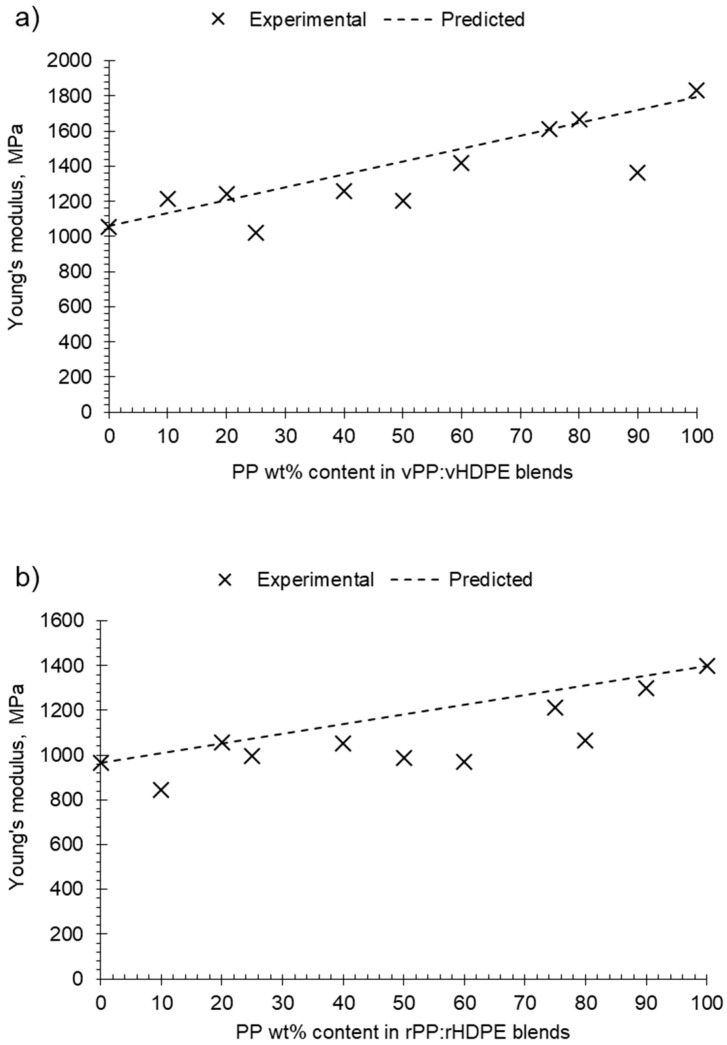
Plot of experimental and predicted Young’s modulus against PP wt% content in PP:HDPE blends: (**a**) vPP:vHDPE blends and (**b**) rPP:rHDPE blends.

The virgin and recycled PP:HDPE blends gave intermediary Young’s moduli values between PP and HDPE. Comparing the predicted rules of mixtures to the experimental Young’s modulus shows a negative deviation for most recycled blends (Figure 9b). A negative deviation suggests poor compatibility and weak adhesion between the phases [6]. It is important to note that the rule of mixtures does not take into account interactions between components. The Young’s modulus values of the virgin blends showed positive and negative deviations from the rule of mixtures with composition (Figure 9a): positive deviation for blends vP10 and vP20, negative deviation between vP25 and vP60, a minor positive deviation at vP75 and vP80, and a negative deviation at vP90. Lovinger and Williams [82] observed a maximum deviation at P80 and suggested that PE can play the role of stiffener to the PP matrix; in sufficient quantities, it enhances the intercrystalline links. The alternating changes of positive and negative deviations suggest a complex interplay of morphological factors and crystallinity as the composition varied. 

## 4. Conclusions

This study investigated the thermal and mechanical properties of virgin and recycled PP:HDPE blends. Thermal studies carried out by DSC confirmed that both the virgin and recycled PP:HDPE blends were immiscible. The recycling process was found to lower the *T*_m_ values of the rPP:rHDPE blends due to structural deterioration and the formation of imperfect crystallites. Interestingly, there was little difference in the *T*_c_ values when comparing the virgin and recycled blends. The PP and HDPE crystallinities were dependent upon the blend composition. As the ratio of PP increased, the crystallinity of PP increased and that of HDPE decreased in the PP:HDPE blends. Generally, the rPP:rHDPE blends had a lower overall crystallinity compared with the vPP:vHDPE blends, suggesting the formation of imperfect crystallites and a heterogeneous crystalline morphology. However, the crystallisation of the individual polymers was more complex. vPP crystallinity was enhanced (compared with rPP) at higher PP content; conversely, rHDPE crystallinity was enhanced (compared with vHDPE) at higher PP content. rPP and rHDPE could contain contaminants due to the difficulty of separating PP and HDPE during the recycling process, thus affecting the crystallinity behaviour.

DMA analysis found little variation in the *E*′ of the virgin and recycled blends with composition. However, a decreasing trend was observed for the virgin blends *E*″ as the PP wt% increased, while the recycled blends’ *E*″ was relatively constant. The interplay between the structure and dissipation mechanisms can be complex. Chain scission caused by the recycling process can introduce plasticising shorter chains. However, impurities can act as fillers in the recycled materials that impose a mechanical restraint. Recycled blends were found to have lower *T*_α_ and *T*_β_ due to structural deterioration caused by the recycling process. Recycled blends gave a reduced Young’s modulus and yield strength in comparison with virgin blends due to deterioration during the recycling process. Generally, the recycled blends gave a higher elongation at break compared with the virgin blends, possibly due to the plasticity effect of the low-molecular-weight chain fragments. However, a comparison between the virgin and recycled blends’ elongation at break was not straightforward in all cases due to the highly orientated vHDPE induced by the injection moulding.

This work explored the variability in the thermomechanical behaviour of vPP:vHDPE and rPP:rHDPE blends without the addition of other components. Understanding the performance variability in recycled blends is key to increasing the quantity of recycled material re-entering the consumer market to contribute towards a circular plastic economy.

## Figures and Tables

**Table 1:** Melting and crystallisation behaviour of vPP:vHDPE and rPP:rHDPE blends obtained from the first heating and cooling cycles using DSC. Bold values are for vPP:vHDPE blends and un-bolded values are for the rPP:rHDPE blends. Related graphs are presented in the Supporting Information (Figures S1–S5).

PP wt% Content in PP:HDPE	Peak Melting Temperature (°C)		Enthalpy of Fusion (J g^−1^)		Peak Crystallisation Temperature (°C)		Enthalpy of Crystallisation (J g^−1^)	Percentage Crystallinity (%)	
	PP	HDPE	PP	HDPE	PP	HDPE	(PP + HDPE)	PP	HDPE
0	**-**	**134.6**	**0.0**	**161.2**	**-**	**118.4**	**161.4**	**0.0**	**56.2**
	159.0	132.9	11.8	115.4	125.0	116.6	147.4	7.3	36.2
10	**173.9**	**132.6**	**3.8**	**161.0**	**-**	**115.6**	**161.6**	**1.8**	**56.1**
	166.4	131.7	27.3	87.8	122.5	118.0	131.4	12.5	30.8
20	**166.1**	**130.5**	**11.4**	**105.8**	**-**	**115.9**	**159.7**	**5.5**	**36.9**
	166.1	131.5	18.9	87.0	123.1	118.4	137.3	14.0	30.4
25	**165.8**	**130.9**	**12.9**	**114.2**	**-**	**116.4**	**148.3**	**6.2**	**36.8**
	165.2	131.5	27.2	72.4	123.4	117.7	116.5	13.9	23.6
40	**167.6**	**130.5**	**31.1**	**78.5**	**124.5**	**117.1**	**131.4**	**15.0**	**27.4**
	163.2	131.3	27.6	79.5	122.0	118.4	129.3	12.0	27.2
50	**172.8**	**134.6**	**41.5**	**79.7**	**126.5**	**117.5**	**110.4**	**20.1**	**27.8**
	162.2	130.7	30.3	68.6	122.4	117.9	125.8	13.6	21.9
60	**165.9**	**127.9**	**38.2**	**55.2**	**124.1**	**117.7**	**117.4**	**18.4**	**19.2**
	162.7	130.8	21.2	60.6	123.1	118.0	116.3	15.7	20.2
75	**168.5**	**128.9**	**50.0**	**36.6**	**123.6**	**116.9**	**108.6**	**24.2**	**12.7**
	164.6	130.5	37.5	50.0	122.0	118.5	110.5	16.9	16.4
80	**168.0**	**128.7**	**55.2**	**24.0**	**124.4**	**116.9**	**94.5**	**26.7**	**8.4**
	163.2	130.2	31.4	53.7	122.2	118.1	105.3	15.6	17.7
90	**168.2**	**128.7**	**66.9**	**5.4**	**121.8**	**116.1**	**88.6**	**32.3**	**1.9**
	161.8	130.3	32.7	41.0	121.8	117.8	107.1	17.0	13.9
100	**167.4**	**-**	**64.4**	**0.0**	**122.3**	**-**	**83.0**	**31.1**	**0.0**
	164.9	129.5	42.8	31.1	122.7	118.2	100.2	19.6	10.6

## Data Availability

Data available upon request.

## Supplementary Materials

The following supporting information can be downloaded at: https://www.mdpi.com/article/10.3390/polym15214200/s1, Table S1: Summary of the crystallinities obtained from the first and second heating ramps for vPP:vHDPE blends; Table S2: Summary of the crystallinities obtained from the first and second heating ramps for rPP:rHDPE blends; Table S3: Summary of *T*_α_ and *T*_β_ taken from tanδ traces for virgin and recycled PP:HDPE blends; Figure S1: Melting temperatures of vPP, rPP, vHDPE and rHDPE of vPP:vHDPE and rPP:rHDPE blends obtained from DSC; Figure S2: Enthalpy of fusion of vPP, rPP, vHDPE and rHDPE of vPP:vHDPE and rPP:rHDPE blends obtained from DSC; Figure S3: Crystallisation temperatures of vPP, rPP, vHDPE and rHDPE of vPP:vHDPE and rPP:rHDPE blends obtained from DSC; Figure S4: Enthalpy of crystallisation of vPP:vHDPE and rPP:rHDPE blends obtained from DSC; Figure S5: Percentage crystallinity of vPP, rPP, vHDPE and rHDPE, and total crystallinity of vPP:vHDPE and rPP:rHDPE blends obtained from DSC.
