# Supplementary material for: Thermomechanical Properties of Virgin and Recycled Polypropylene—High-Density Polyethylene Blends
Source: Polymers (Basel). 2023 Oct 24;15(21):4200. doi: 10.3390/polym15214200 (PMC10647352; doi:10.3390/polym15214200)
Supplement: Supplementary file 1 [file polymers-15-04200-s001.zip › polymers-2615102-supplementary.pdf]

<u>Supporting Information</u>

Thermomechanical Properties of Virgin and Recycled Polypropylene – High-Density Polyethylene Blends

Hannah Jones[1], Jake McClements[2], Dipa Ray[1], Colin S. Hindle[3], Michail Kalloudis[4], and Vasileios Koutsos[1,]*

[1]School of Engineering, Institute for Materials and Processes, The University of Edinburgh, Sanderson Building, King's Buildings, Edinburgh EH9 3FB, United Kingdom

[2]School of Engineering, Newcastle University, Merz Court, Claremont Road, Newcastle Upon Tyne NE1 7RU, United Kingdom

[3]School of Engineering and the Built Environment, Edinburgh Napier University, Merchiston Campus, 10 Colinton Road, Edinburgh EH10 5DT, United Kingdom

[4]Impact Laboratories Ltd. (Impact Solutions), Impact Technology Centre, Fraser Road, Kirkton Campus, Livingston EH54 7BU, United Kingdom

*Corresponding author, e-mail: vasileios.koutsos@ed.ac.uk, Tel +44 (0)131 650 8704

Table S1: Summary of the crystallinities obtained from the first and second heating ramps for vPP:vHDPE blends.

PP wt% in vPP:vHDPE blends	First Heat Crystallinity, %		Second Heat Crystallinity, %	
	PP	HDPE	PP	HDPE
0	0.0	56.2	0.0	59.2
10	1.8	56.1	1.6	55.2
20	5.5	36.9	5.8	45.4
25	6.2	39.8	6.6	42.3
40	15.0	27.4	13.3	28.2
50	20.1	27.8	12.5	25.0
60	18.4	19.2	20.2	20.3
75	24.2	12.7	27.1	10.5
80	26.7	8.4	28.8	7.0
90	32.3	1.9	32.1	2.6
100	31.1	0.0	42.2	0.0

Table S2: Summary of the crystallinities obtained from the first and second heating ramps for rPP:rHDPE blends.

PP wt% in rPP:rHDPE blends	First Heat Crystallinity, %		Second Heat Crystallinity, %	
	PP	HDPE	PP	HDPE
0	5.7	40.2	7.3	36.2
10	13.2	30.6	12.5	30.8
20	9.1	30.3	14.0	30.4
25	13.1	25.2	13.9	23.6
40	13.3	27.7	12.0	27.2
50	14.7	23.9	13.6	21.9
60	10.2	21.1	15.7	20.2
75	18.1	17.4	16.9	16.4
80	15.1	18.7	15.6	17.7
90	15.8	14.3	17.0	13.9
100	20.7	10.8	19.6	10.6

Table S3: Summary of T_α and T_β taken from tanδ traces for virgin and recycled PP:HDPE blends.

PP wt% in PP:HDPE blends	Virgin		Recycled	
	T_α	T_β	T_α	T_β
0	115.9	-	106.7	4.6
10	113.8	-	109.0	3.8
20	110.6	-	108.3	6.7
25	101.1	12.4	101.7	8.5
40	102.2	12.6	96.8	5.6
50	98.2	10.2	105.2	3.6
60	98.9	11.6	99.1	6.4
75	95.8	15.4	97.1	4.5
80	99.1	15.5	93.4	4.1
90	95.2	11.2	90.1	5.2
100	93.5	8.9	88.1	5.0

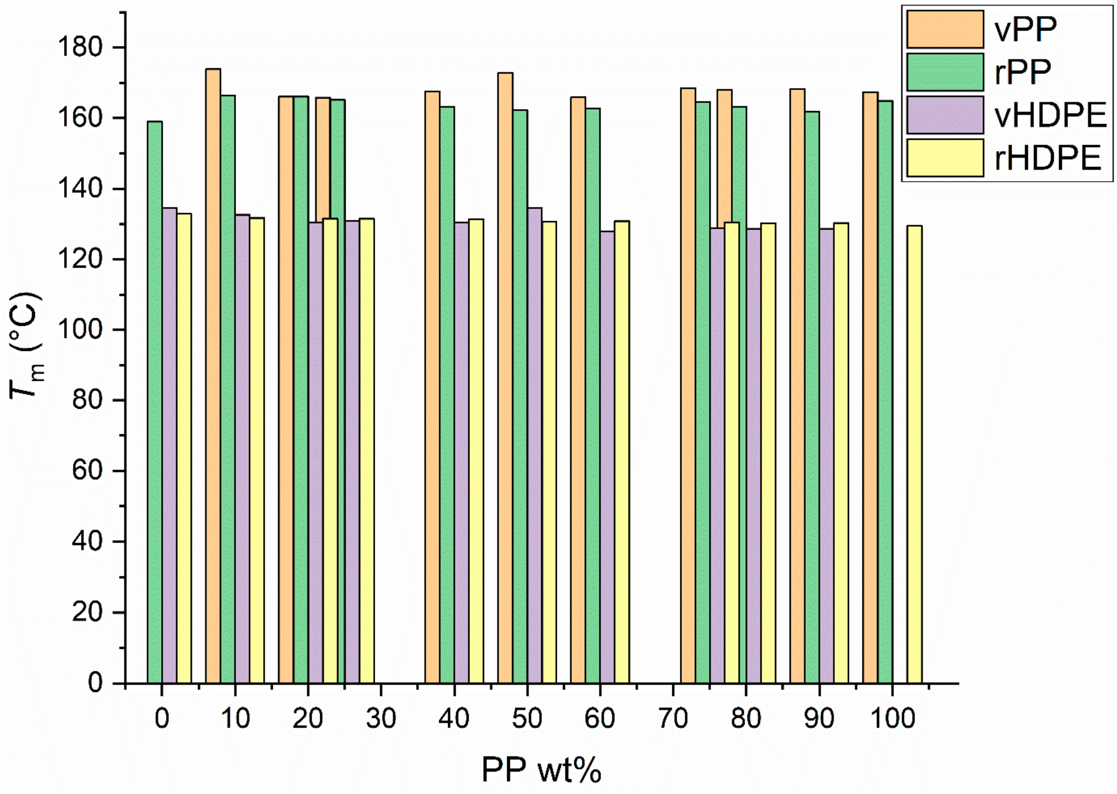

Figure S1. Melting temperatures of vPP, rPP, vHDPE, and rHDPE of vPP:vHDPE and rPP:rHDPE blends obtained from DSC.

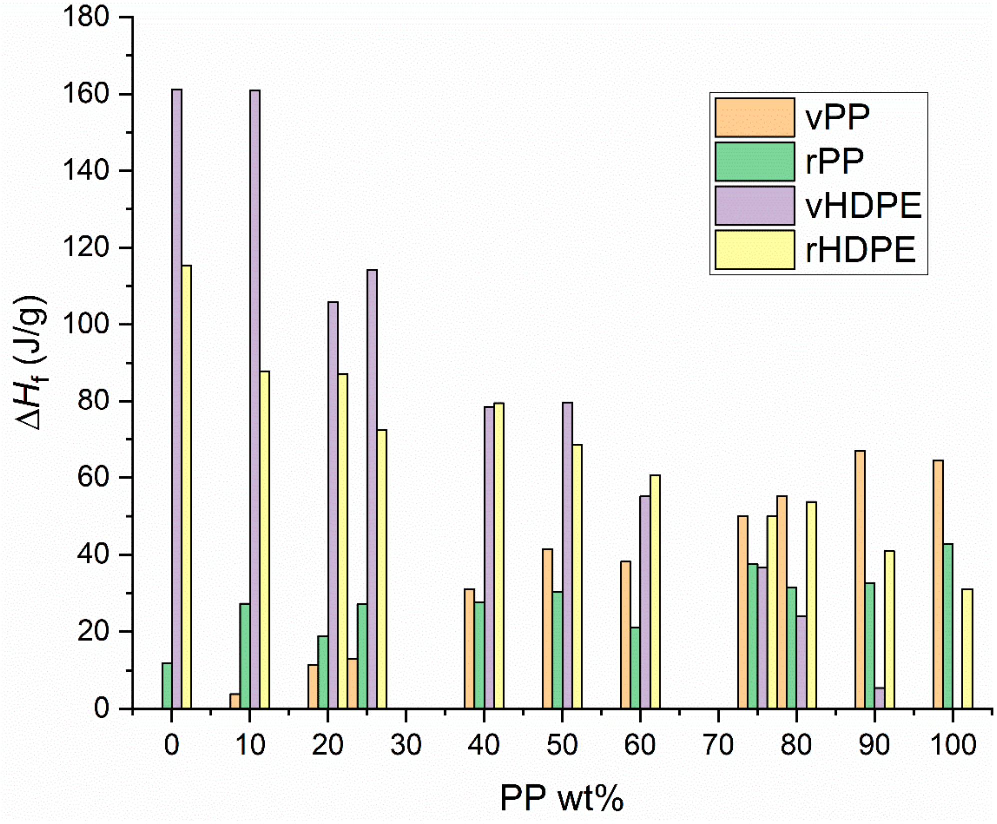

Figure S2. Enthalpy of fusion of vPP, rPP, vHDPE, and rHDPE of vPP:vHDPE and rPP:rHDPE blends obtained from DSC.

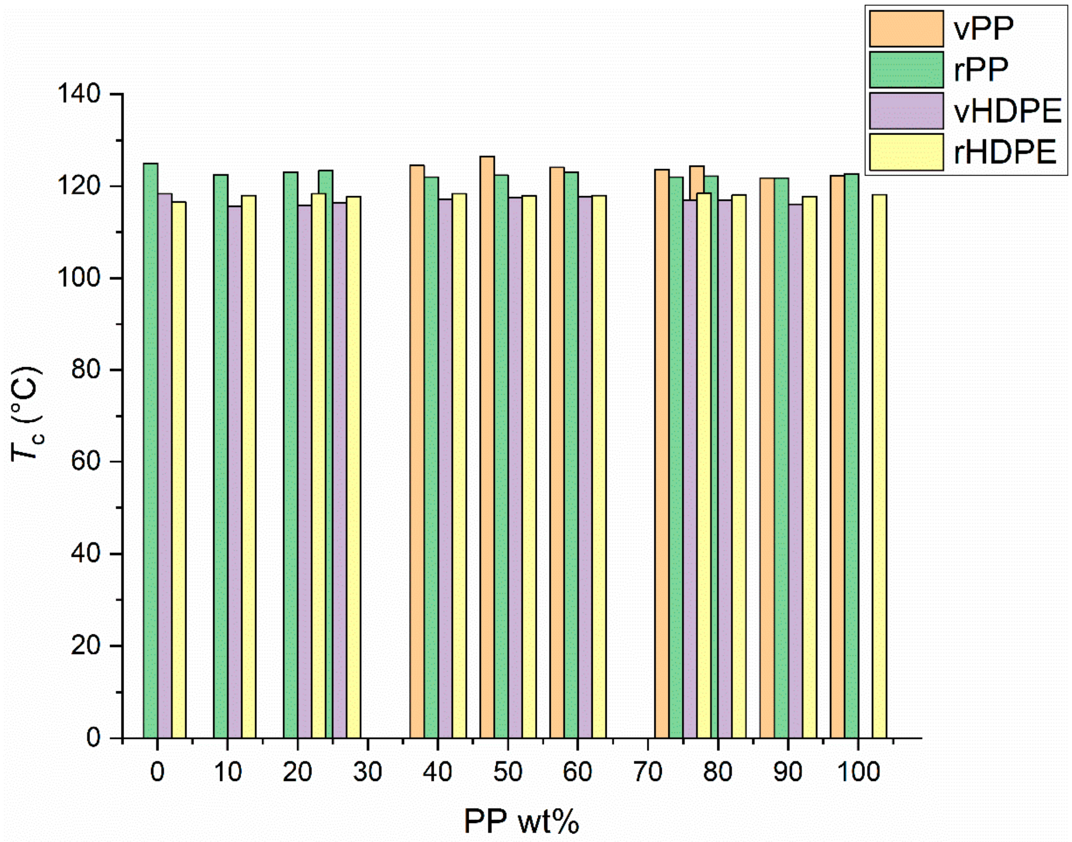

Figure S3. Crystallisation temperatures of vPP, rPP, vHDPE, and rHDPE of vPP:vHDPE and rPP:rHDPE blends obtained from DSC.

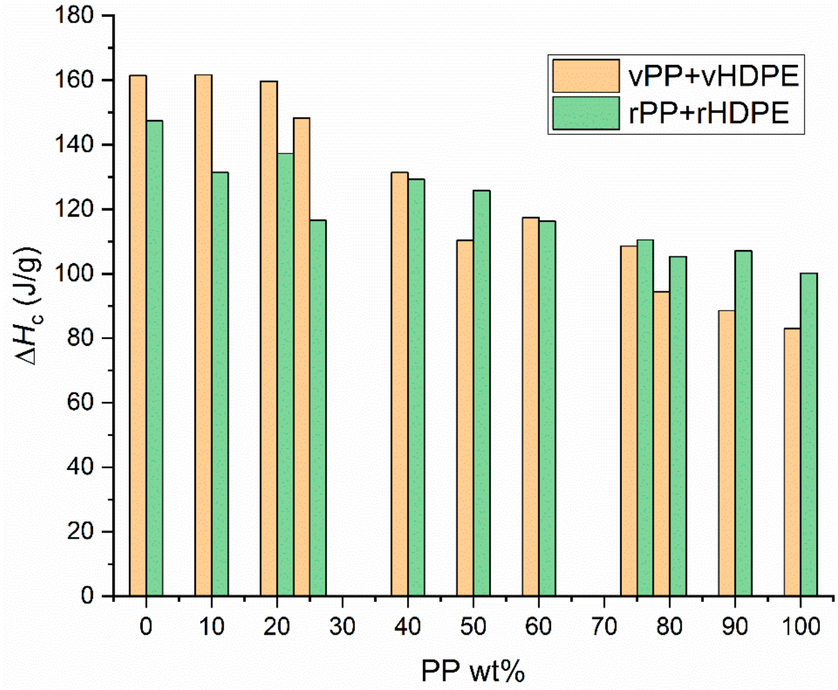

Figure S4. Enthalpy of crystallisation of vPP:vHDPE and rPP:rHDPE blends obtained from DSC.

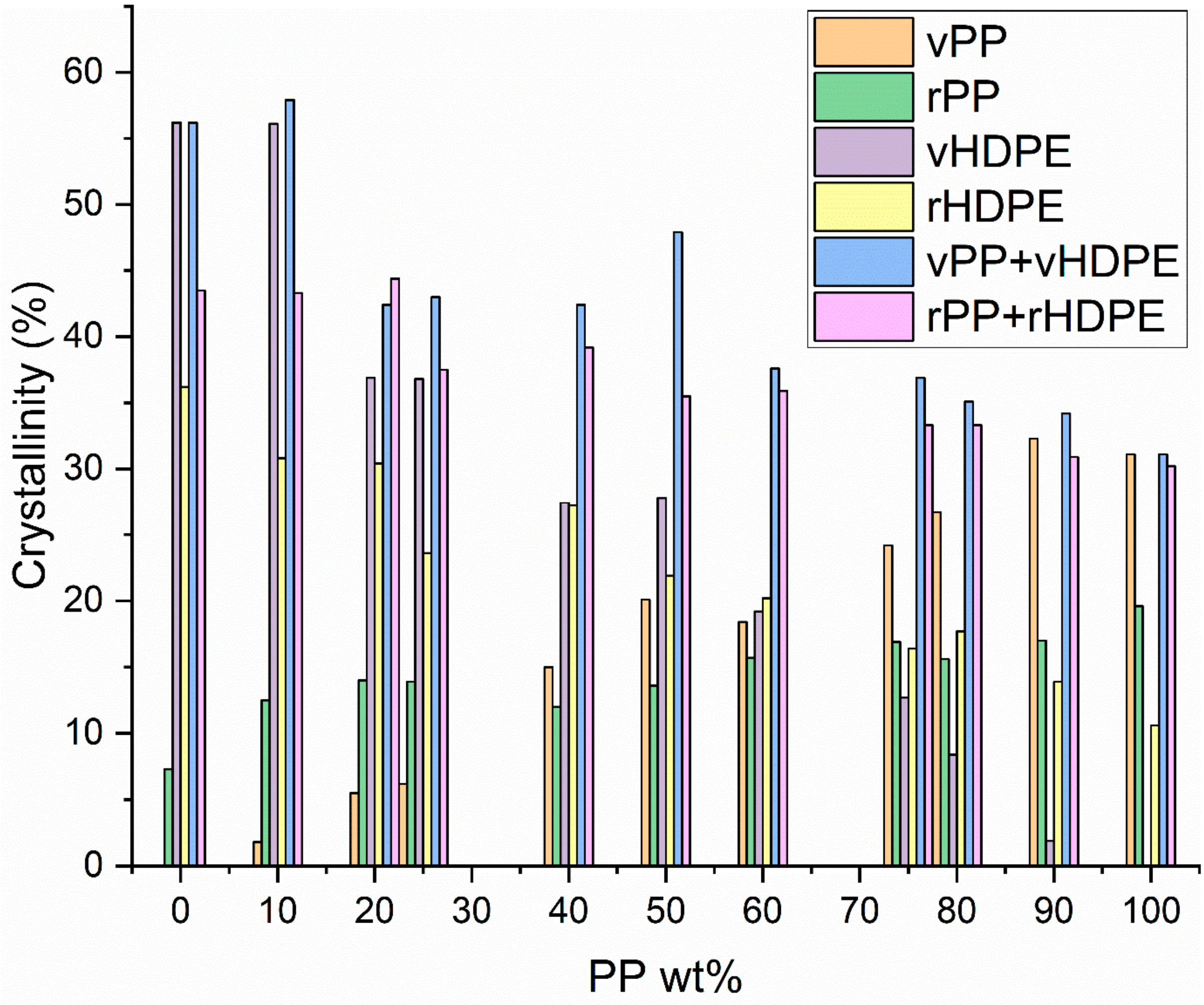

Figure S5. Percentage crystallinity of vPP, rPP, vHDPE, rHDPE, and total crystallinity of vPP:vHDPE and rPP:rHDPE blends obtained from DSC.
